# A Novel Classification Technique of Arteriovenous Fistula Stenosis Evaluation Using Bilateral PPG Analysis

**DOI:** 10.3390/mi7090147

**Published:** 2016-08-23

**Authors:** Yi-Chun Du, Alphin Stephanus

**Affiliations:** Department of Electrical Engineering, Southern Taiwan University of Science and Technology, Tainan City 71005, Taiwan; peppymusila52@gmail.com

**Keywords:** arteriovenous fistula (AVF) stenosis, bilateral photoplethysmography (PPG), error correcting output coding support vector machine-one versus rest (ESVM-OVR), artificial neural network (ANN), degree of stenosis (DOS), positive predictive value (PPV)

## Abstract

The most common treatment for end-stage renal disease (ESRD) patients is the hemodialysis (HD). For this kind of treatment, the functional vascular access that called arteriovenous fistula (AVF) is done by surgery to connect the vein and artery. Stenosis is considered the major cause of dysfunction of AVF. In this study, a noninvasive approach based on asynchronous analysis of bilateral photoplethysmography (PPG) with error correcting output coding support vector machine one versus rest (ESVM-OVR) for the degree of stenosis (DOS) evaluation is proposed. An artificial neural network (ANN) classifier is also applied to compare the performance with the proposed system. The testing data has been collected from 22 patients at the right and left thumb of the hand. The experimental results indicated that the proposed system could provide positive predictive value (PPV) reaching 91.67% and had higher noise tolerance. The system has the potential for providing diagnostic assistance in a wearable device for evaluation of AVF stenosis.

## 1. Introduction

Recently more than 660,000 American people are kidney failure patients, commonly known as end-stage renal disease (ESRD), including 468,000 patients on dialysis treatment. This was reported by the US Renal Data System in its annual report [1]. The most frequent treatment used by patients with ESRD is hemodialysis (HD). In the HD process, a dialyzer is vital equipment to purify the blood of unused products and excess fluid. A needle from the dialyzer with vascular access is usually inserted into the forearms of the patient to reach the blood. Placement of connection points, which is also called anastomosis, is usually close to the patient’s wrist or elbow. Generally, this is the way a vascular access, called an arteriovenous fistula (AVF), is created and done by a surgical operation in which arteries and veins are connected together. As mentioned in another study [2,3], AVF provides the lowest risk of death caused by inadequate vascular access, low complication rate, long term use, and lower cost compared to other (arteriovenous graft and central venous catheter) vascular access options. Ready to use (mature) AVF are characterized by the formation of venous distension and arterialization after 6–12 weeks. Usually, each HD patient requires about 4 h and is done three times per week for the rest of the patient life unless he or she is able to get a kidney transplant. Repeated puncturing in the long term usually leads to access occlusion (stenosis) and failures which are caused by inadequate arterial inflow or venous outflow. This causes thrombosis, resulting in intimal hyperplasia, chronic fibrin, cellular deposit, aneurysm, and limb ischemia [4,5,6]. Thus, vascular access evaluation and monitoring may help HD patients to avoid stenosis and thrombosis.

Some of the current research seeks to measure and evaluate AVF stenosis by various methods. Bash et al. in 2005 argued that angiography is often used to evaluate the level of clinical vascular stenosis [7]. Nevertheless, angiography has disadvantages, such as being invasive, requiring surgery, and radiation exposure. Furthermore, using Doppler ultrasound, the diameter of a vascular stenosis can be evaluated [8]. The drawback of these two methods is that their equipment prices are economically expensive and need a person who has special expertise to operate them. In 2009, Vasquez et al. used wavelet and support vector machines to detect AVF stenosis by the sound of blood flow [4]. Wang et al. in 2014 have improved the sound features of AVF blood flow stenosis by experiments exploiting the frequency and time domain analysis [9]. In 2015, Wu et al. measured the difference of blood volume changes between the right and left side of the PPG signal by utilizing a self-synchronization error formulation (SSEF). The degree of stenosis (DOS) can be expressed by a significant difference in rise time (RT) and amplitude (AMP) on bilateral PPG [10]. In clinical research, DOS has been demonstrated in numerous studies [6,10,11,12] and became an appropriate reference to grade AVF stenosis. The pulse of bilateral PPG will progressively come to be asynchronous to every single heartbeat if vascular access turns into an obstruction on one side of the arm. Furthermore, arteriovenous access has two significant conditions, which are stenosis of inflow or outflow and intra-flow decreases [13]. There are many features that have been experimented to detect AVF stenosis from bilateral PPG, including RT, AMP, and pulse transit time (PTT) [14].

To acquire the changes of blood volume non-invasively in the tissue micro vascular bed, as a technique of optical measurement, PPG is an appropriate option. The waveform of PPG conveys some specific physiological information, such as respiration, vasomotor activity, thermoregulation, and the changes of cardiac synchronicity in the volume of blood with each heartbeat [15]. The PPG signal was taken at the thumbs, index finger, great toe, and ear lobe sites using multi-channel data measurement. In addition, the results of these measurements can be used as input for a digital signal processing-based computer and analysis of the pulse wave. [16]. It can be argued that this technique is a suitable solution for monitoring and evaluation of AVF stenosis.

As mentioned in [6,10,11,12], DOS is an important parameter to help surgeons grade (classify) the severe of vascular access. To classify AVF stenosis associated with its DOS a novel noninvasive technique was executed in this study based on bilateral PPG analysis. Here, the features of AVF stenosis were measured and calculated from bilateral differences (asynchronous) of the PPG signal, which were taken from left and right thumbs in the time domain. Error correcting output coding support vector machine one versus rest (ESVM-OVR) is exploited to determine the degree of AVF stenosis. To confirm the performance and reliability of the proposed technique, an ANN back-propagation technique and noisy signal which have several signals to noise ratio (SNR) is utilized and compared. Verifying the ESVM-OVR for AVF stenosis classification based on bilateral PPG signals analysis is a noticeable contribution of this present study.

## 2. Material and Method 

### 2.1. Material

**Figure 1:**
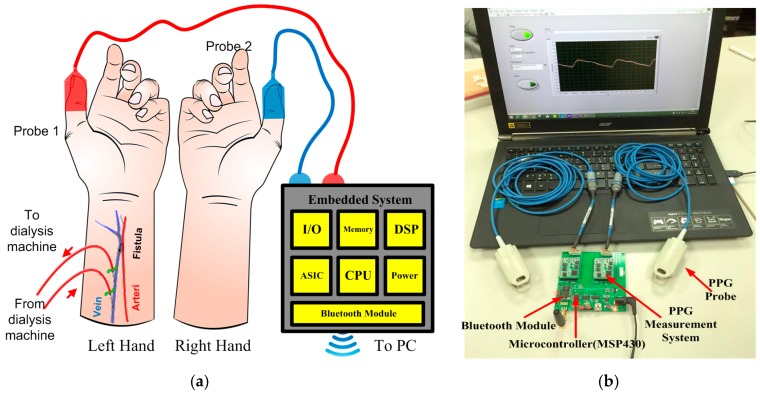
Monitoring and recording system: (**a**) PPG probes placement; and (**b**) embedded system connected to a laptop computer.

In this study, the system is planned to record and monitor the change of blood volume between the left and the right (bilateral) thumb site on the hand. The probe was mounted within the clip at the left and the right thumb to acquire bilateral PPG signals, as shown in Figure 1a. For the analysis requirements, two-channel PPG signals at a sampling rate of 1 kHz are captured synchronously. This signal is then inputted to the embedded system (MSP430, Texas Instruments, Dallas, TX, USA) to provide analog to digital conversion of data and transfer it to a laptop computer with MATLAB (ATA Engineering Inc., San Diego, CA, USA) as the analyzing tool. In a sampling window, the interval of pulse foot (PF)–pulse foot (PF) is also located by the MSP430 to obtain sampling data. The proposed system can provide a promising continuous measurement during hemodialysis treatment, as shown in Figure 1b. The training process and the classifier architecture are two essential parameters to design a classifier. In this study neural network and ESVM-OVR techniques were implemented by MATLAB 8.3-R2014a software.

### 2.2. Method

#### 2.2.1. Preprocessing (Step 1–2)

The raw data has to be preprocessed before extracting the bilateral PPG signal features. First, a second-order of infinite impulse response (IIR)-Butterworth low pass filter (LPF) with a 20 Hz frequency cut off is employed to filter the raw data, which removes noise due to physiological signals and motion artifacts.

#### 2.2.2. Feature Extraction (Step 3–5)

**Figure 2:**
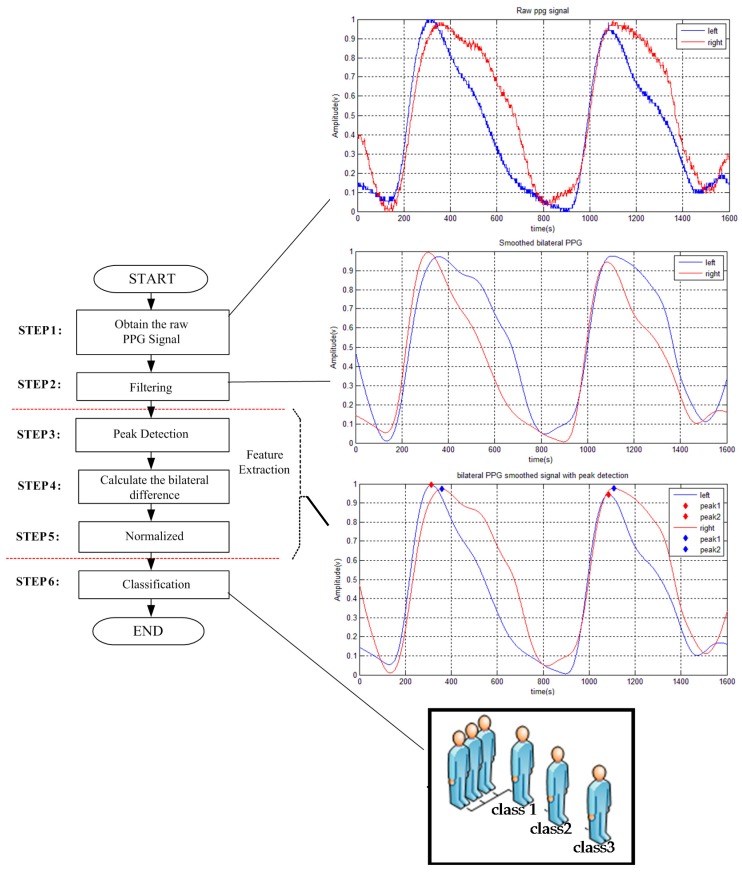
The method of the proposed system.

The increasing vascular resistance or occlusion causes changes to the PPG pulse shape and the transit time will be extended. An asynchronous PPG pulse appears at the left and right sites as can be seen in Figure 2. To accomplish this task, establishing a reference from each peak of two pulses that are found in the sampling window, which consists of a dicrotic notch and systolic rising edge area, is needed. The bilateral difference (*d_i_*) is defined as follows:

(1) $$d_{i}=\left|x_{\text{Ri}}-x_{\text{Li}}\right|,i=1,2,3,\ldots n$$

The sampling data that are $x_{\text{Ri}}$ and $x_{\text{Li}}$ are obtained from the left and right PPG signal, consecutively. To calculate the difference pattern Φ is used, in which Φ = [*d*_1_, *d*_2_, *d*_3_,…*d_i_*,…*d_n_*]. A feature with a value which lies in a different range was a common problem in the feature extraction field. Here, a linear normalization can be expressed (Equation (2)):

(2) $$\hat{x_{ik}}=\frac{x_{ik}-\text{min}\left(x_{k}\right)}{\text{max}\;\left(x_{k}\right)-\text{min}\left(x_{k}\right)}$$

The greater the increase in the time delay, the greater the increase in the severity of the disease. Corresponding to these patterns, we could systematically build comparative feature patterns which are composed by 88 vectors (22 vectors obtained from 22 subjects obtained from three classes) of four dimensions (the extracted feature vectors dimension). The extracted feature of the PPG signal is determined using the term vector. The mean of those subjects were 69.4 ± 13.1 years and recruited from the Kaohsiung Veterans General Hospital (KVGH)-Institutional Review Board (IRB), Tainan Branch and VGHKS13-CT12-11 as the contract number. In clinical research terms, DOS is a degree index of the narrowing of the normal vessel of AVF subjects and it is measured by B-mode ultrasound or angiography images. These subjects were divided according to their DOS severity class. These experiments followed IRB approval procedures. The method of the proposed system is presented in Figure 2.

**Figure 3:**
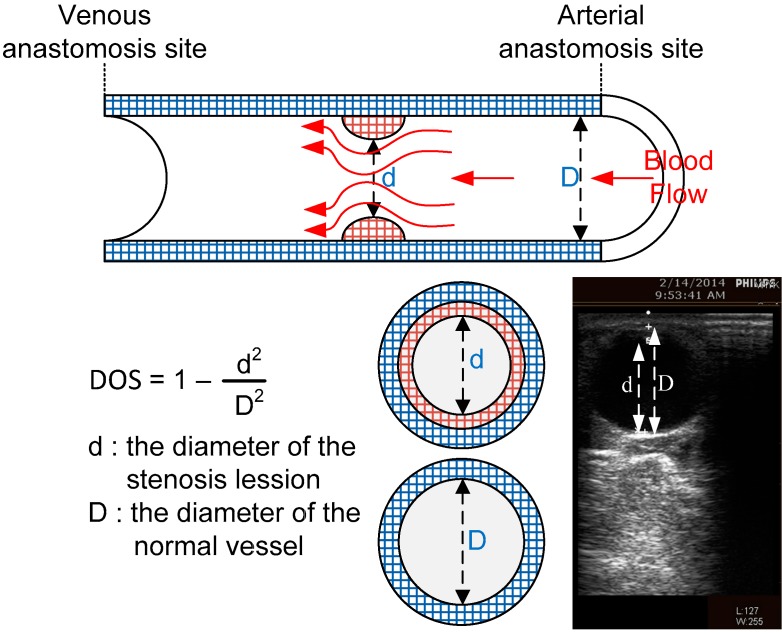
Measurement area of the degree of stenosis (DOS) component with respect to the B-mode ultrasound image.

Referring to previous research [13], DOS has become a main reference to grade vascular disease as severe and this can be expressed as $\text{DOS}\%=(1-\frac{d^{2}}{D^{2}})\times 100\%$, where *d* is the stenosis lesion diameter and *D* is the normal vessel diameter in the blood flow direction. Figure 3 shows the area of *d* and *D*. If DOS is 100%,this means total occlusion, over 50% usually means the patient requires surgical treatment, and between 30% and 50% means it may have an effect upon the efficiency of hemodialysis. As a result, we followed clinical comments to design three different classes for classification as shown in Table 1.

#### 2.2.3. Classification (Step 6)

(a) Support Vector Machine

Vapnik was the first to introduce the support vector machine (SVM), which works on the principle of structural risk minimization (SRM) with the goal of finding the “best” hyperplane that separates two classes in the input space [17,18,19]. A typical characteristic of SVM is obviously seen in its ability to map complicated data to a feature space of high dimension by the non-linear mapping of the target vector and input vector. For a classification problem, the adaptive capability will decrease, and training time will increase, when a combination of individual classifiers with training patterns are used. Therefore, the SVM should be transformed to a linear machine which labels the input pattern as one class or none, and one output that is capable of handling non-linearity and linearity, which is separable for two class training patterns. Henceforth, this optimum hyperplane is used to achieve binary classification.

Training, in terms of the SVM, is a quadratic optimization problem. The hyperplane formation *w^T^x* + *b* = 0 (*w* is the hyperplane coefficients vector and *b* is a bias term) then maximizes the margin between the hyperplane and the nearest point that could be used as the quadratic optimization problem. It has been proven by many researchers that SVM provides high generalization ability. For a binary classification problem, in the feature space that has formed from the optimum hyperplane, an unknown pattern *y* will be built and obtain its decision algorithm from:

(3) $$f\left(y\right)=sgn\left(\overset{N}{\underset{i=1}{\sum }}\alpha_{i}y_{i}K\left(x_{i},y\right)+b\right)$$

where α*_i_* ≥ 0, *i* = 1,2,…, *N* are non-negative Lagrange multipliers which fulfill $\sum_{i=1}^{N}\alpha_{i}y_{i}=0$, the class labels defined as ${\left\{y_{i}|y_{i}\in \left\{-1,+1\right\}\right\}}_{i=1}^{N}$, ${\left\{x_{i}|x_{i}\in R^{N}\right\}}_{i=1}^{N}$ are the training patterns, and *K*(*x_i_*, *y*) for *i* = 1,2,…, *N* stand for the kernel function of symmetric positive-definite matrix, which explains an inner product in the feature space. A linear combination of the kernels or inner product is defined as *f*(*y*) in this definition. With the kernel function, operations to be performed on the input space are made possible in a high-dimensional feature space. There are several kernel functions commonly used in SVM classifiers as shown in Table 2.

Two conditions which should be fulfilled by a kernel function in order to map the input to a high dimension of feature space are Karush–Kuhn–Tucker (KKT) and Mercers condition [19]. In this study, the option to use the proper kernel function is studied empirically and the optimal output is developed using quadratic kernel function.

(b) ESVM-One Versus Rest (OVR)

**Figure 4:**
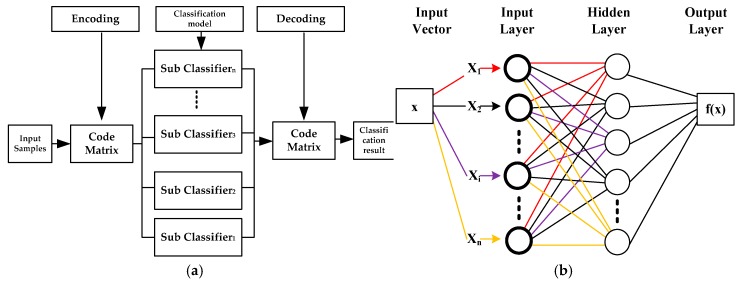
The block diagram of the models: (**a**) Error correcting output coding support vector machine-one versus rest (ESVM-OVR) and (**b**) artificial neural network (ANN).

In this study, error-correcting output coding (ECOC) has been adopted from digital communication theory to be fused with SVM to produce a decision [20]. In the ECOC terminology, several SVM are trained with the limit up to 2^*n*−1^−1 (*n* is the class number) and the main aim of each SVM is to separate different class combinations. It adopts three classifiers for classifying three (X–Z) classes; the first SVM classifier will classify X from the rest (Y and Z), the second SVM classifier will classify Y from the rest (X and Z), and the last SVM classifier will classify Z from the rest (X and Y). An unknown pattern can obtain its multiclass classifier output code by the combination of all separate SVM targets. The example classes X, Y, and Z have codes (1, 1, 1), (0, 1, 0), and (0, 0, 1), respectively. The multiclass classifier target code is met, when every one of the separate SVMs classifies a pattern correctly and the ECOC will report no error. However, an incorrect decision occurs when at least one of the SVMs misclassifies the pattern. The class chosen for this pattern is one of the closest targets codes in the Hamming distance [21]. The ECOC will reclassify the training sample, using the values of the code bit in the error-correcting code matrix columns. Then several unrelated binary-class sub-classifiers are built. A typical decoding strategy is then used to classify the sample. The ECOC schematic and code matrix is used in this study as shown in Figure 4a and Table 3, respectively.

(c) Artificial Neural Network

Inspired by the biological nervous system cells, an ANN is a mathematical structure with a flexible pattern. A common ANN structure, called a multilayer perceptron network (MLPN), is composed of input, hidden, and output layers with their nodes and activation functions. Recently, in science and technology, and also in various branches such as chemistry, physics, and biology, artificial neural networks (ANNs) applications are widely used. One hidden layer trained by back-propagation is used to build the MLPN model in this study and its schematic diagram is shown in Figure 4b. The activation function consists of a log-sigmoid function in the hidden layer and a linear function in the output layer.

## 3. Results

### 3.1. Feature Extraction

**Figure 5:**
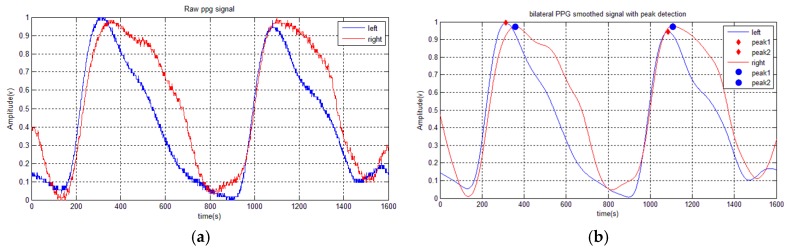
Monitoring and recording system: (**a**) PPG probes placement; and (**b**) the embedded system connected to a tablet PC.

The proposed AVF stenosis classifier was built on a laptop computer Intel Core i5 1.8 GHz with 12 GB of RAM. The performance of the proposed technique is examined with four parameters, such as sensitivity, specificity, precision, and accuracy. The bilateral difference is extracted using timing parameters with PPG analysis. In this case, the DOS will become the main reference to classify AVF stenosis. By analyzing the bilateral differences, the timing parameters d1–d4 increased with its occlusion. In addition, the detail data of the subject is shown in Table 4. Therefore, these parameters could be used to classify AVF stenosis. Furthermore, the main rule of classification for the three classes is based on considerable amount of examination and the comment of professional physician. Figure 5 shows the result of the feature extraction stage, from the raw data to the smoothed bilateral PPG with peak detection.

**Figure 6:**
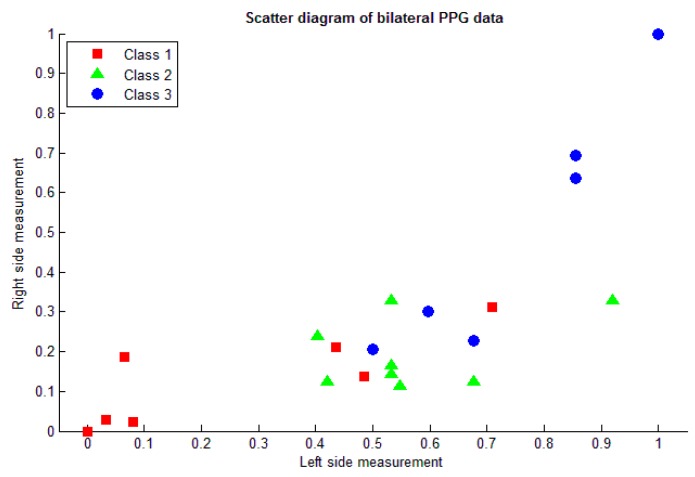
Scattered plot of bilateral PPG feature.

Table 4 presents the result of the DOS calculation with respect to the values of *D* and *d* measurement. The ranges of difference patterns after normalization calculation ware 0.00–0.71, 0.11–0.92, and 0.14–1, divided into three classes, respectively. The results of all bilateral PPG features are then plotted as shown in Figure 6.

### 3.2. Classification

The two distinctive factors influence the generalization capability of the SVM is controlled by, firstly, the capacity of the learning machine measured by its Vapnik–Chervonenkis (VC) dimension and, secondly, by the training error rate [19]. In order to get confirmation of the optimum result, the SVMs compare their performance and are trained for several kernels. Furthermore, the most frequently used feed-forward neural network is the MLPN, which is also compared. To classify the bilateral PPG based on four feature inputs, three outputs, with a single hidden layer (36 hidden neurons) is used. For the classification accuracy consideration, the number of hidden neurons is determined and several experiments are performed during implementation of these classifiers. The Levenberg–Marquardt algorithm is used to train the MLPN. To introduce two important properties, the sigmoidal function is used with the range between zero and one. First, to allow the network to perform complex mappings of input to output vector spaces, the sigmoid is nonlinear. Next, it is differentiable and continuous, which allows the gradient of the error to be used in updating the weights.

The confusion matrix shows the classification results of the classifiers and each cell contains the raw number of exemplars classified for the corresponding combination of desired and actual network outputs. The evaluation performance of the classifiers that are built in this study is determined by the calculation of sensitivity, specificity, precision, and accuracy. Table 5 shows the result comparison between the proposed technique (three common kernel functions) and the ANN.

**Figure 7:**
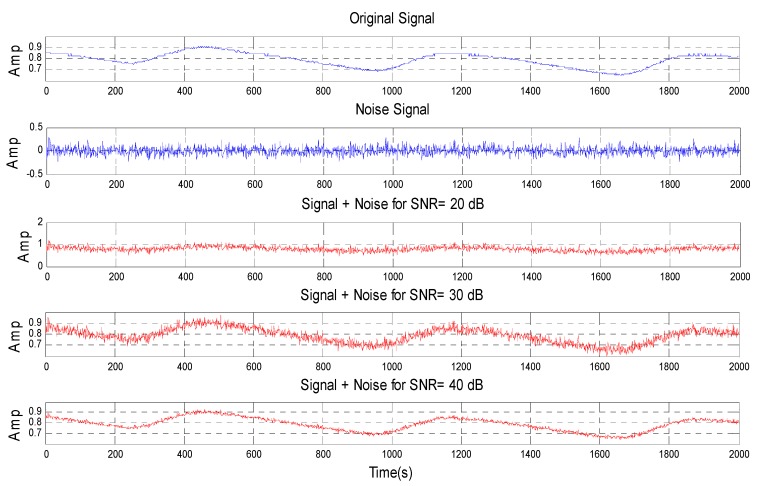
The characteristic of the new data samples.

It can be noticed from the Table 5 that the proposed technique with a quadratic kernel function has the most optimum achievement from other common kernel functions, and even the ANN technique. In this study, the proposed technique with a quadratic kernel function is also examined against noise to derive its performance and reliability. There are twenty data samples (raw PPG signals) that have been predicted 100% correct are chosen to perform this examination. The noise constructed by utilizing those raw PPG signals is manipulated by a certain SNR value. Having formed the noise, adding to the raw PPG signal is the next step to obtain a noisy PPG signal. The new twenty data samples are developed by the collection of the noisy PPG signal. The characteristic of the new data samples in comparison with the raw PPG signal (original signal) and noise signal is presented in Figure 7.

**Figure 8:**
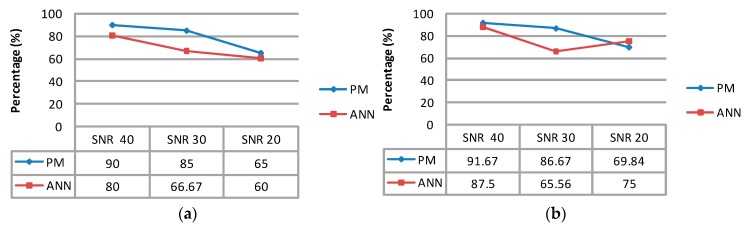

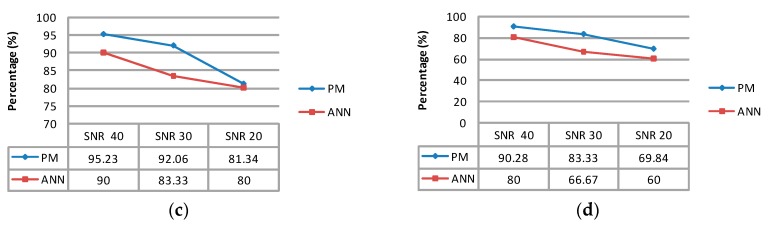
Result comparison of (**a**) accuracy; (**b**) precision; (**c**) specificity and (**d**) sensitivity between the proposed technique and the ANN.

The examining result is shown in Table 6 as follow:

In this step, Table 6 with a SNR = 40 dB indicates that the proposed technique reached its highest level in all statistical parameters. On the other hand, its level declined sharply with a SNR = 20 dB. The proposed technique with twenty new data samples also has been compared with the ANN, as presented in Figure 8. As shown in Table 6 and Figure 8, the proposed technique, in all statistical parameters, has significant predominance against the ANN, which had been evaluated with the noisy PPG signal.

## 4. Discussion

Based on a literature study and the results presented in this study, two stages that could change the classification accuracy in developing automated diagnostic systems are preprocessing and feature extraction. The high classification accuracy of ESVM-OVR gives insights into the features used for classifying AVF stenosis and can also be inferred from the technique that has been built. Bilateral differences are a good feature to separate the severity of the stenosis through the PPG signal of HD patients. Solving quadratic programming problems to find the support vector is the greatest computational effort during the training phase. The SVM maps the features to higher dimensional space and then use an optimal hyperplane in the mapped space. A trial-and-error process was used to select a suitable kernel function. The result of this study demonstrated that ESVM-OVR was significantly satisfying for classifying arteriovenous fistula stenosis in hemodialysis patients. Table 7 shows the experimental comparison of other similar studies to detect AVF stenosis with the proposed technique.

## 5. Conclusions 

In this paper, a multiclass ESVM-OVR is used as a novel classification technique for classifying AVF stenosis based on bilateral PPG. The feature pattern of bilateral PPG signal could be extracted and the patients who have AVF stenosis are graded effectively. The experimental results showed that the proposed technique could properly provide high performance for classifying the AVF stenosis from PPG signal. Moreover, the proposed technique is a good candidate and it has high potential implementation to a portable system as a routine examination device or homecare application.

## Figures and Tables

**Table 1:** Class partition based on DOS.

DOS	Class
DOS ≤ 30%	1
30% ≤ DOS ≤ 50%	2
DOS ≥ 50%	3

**Table 2:** Support vector machine (SVM) kernels and its commonly used function.

Kernel	Kernel Function
Quadratic	$K\left(x,z\right)={\left(x_{i}^{T}z_{i}+\gamma \right)}^{2}$
RBF	$K\left(x,z\right)\;=\text{ exp}\left(-\gamma ∥x_{i}+z_{i}{∥}^{2}\right)$
Linear	$K\left(x,z\right)=x_{i}^{T}z_{i}$

**Table 3:** The errors correcting code matrix for three classes and three code lengths.

Class	ECOC		
	f1	f2	f3
1	1	1	1
2	0	1	0
3	0	0	1

**Table 4:** Subject data.

Class	*d*	*D*	DOS	Age	Gender	
					Male	Female
1	1.52	1.63	0.130415	81	√	
	1.09	1.18	0.146725	78	√	
	1.17	1.36	0.259894	87	√	
	0.94	0.99	0.146725	86	√	
	0.78	0.89	0.145607	65		√
	0.812	0.961	0.34535	81	√	
	0.698	0.797	0.145607	54	√	
	1.45	1.71	0.137399	81	√	
2	0.698	0.797	0.267894	81	√	
	0.78	0.89	0.145665	54	√	
	0.78	0.89	0.352678	67		√
	0.812	0.961	0.268303	87		√
	0.78	0.89	0.278894	84		√
	0.87	0.68	0.353638	83	√	
	0.812	0.961	0.268303	86	√	
	0.812	0.961	0.278894	84	√	
3	0.94	0.99	0.26383	75		√
	0.78	0.89	0.489894	86	√	
	0.812	0.961	0.474949	85	√	
	0.812	0.961	0.474940	89	√	
	0.812	0.961	0.659894	83		√
	0.78	0.75	0.373282	83	√	

**Table 5:** The optimum statistical parameters values of the classifiers and the central processing unit (CPU) times consumed for training.

Classifiers		Performance Parameters				
**Proposed Technique**	**Kernel Function**	**Accuracy (%)**	**Precision (%)**	**Specificity (%)**	**Sensitivity (%)**	**CPU Time (s)**
	**Quadratic**	90.9	92.59	95.23	88.89	0.22
	**Linear**	77.27	82.22	88.09	77.79	0.16
	**RBF**	72.72	85.71	85.71	70.83	0.19
**ANN**		80.00	76.67	91.84	88.89	3.00

**Table 6:** The comparisons of optimum statistical parameters of proposed technique with respect to their noisy signal.

Added Noise	Accuracy (%)	Precision (%)	Specificity (%)	Sensitivity (%)
SNR = 40	90.00	91.67	95.23	90.28
SNR = 30	85.00	86.67	92.06	83.33
SNR = 20	65.00	69.84	81.34	69.84

**Table 7:** Comparing the experimental result with other similar study.

The Approaches	Du et al. [10]	Wang et al. [9]	Wu et al. [14]	Chen et al. [11]	Proposed Technique
The Clinical Stenosis Detector	PPG	Stethoscope	Doppler Ultrasound	Stethoscope	PPG
Classifier Architecture	Cooperative Game Detector	ANN	I-G Decision Making	ANFIS	ESVM-OVR
The Number of Classes	3	2	2	3	3
System Performance Rate–PPV (%)	-	87.84	>80	-	91.67%
CPU Times Rates (seconds)	-	-	-	-	0.22

## Abbreviations

The following abbreviations are used in this manuscript:

ESRD: End state renal disease
HD: Hemodialysis
AVF: Arteriovenous fistula
PPG: Photoplethysmography
ESVM-OVR: Error correcting output coding support vector machine-one versus rest
DOS: Degree of stenosis
ANN: Artificial neural network
PPV: Positive predictive value
PF: Pulse foot
IIR: Infinite impulse response
LPF: Low pass filter
SRM: Structural risk minimization
KKT: Karush-Kuhn-Tucker
ECOC: Error correcting output coding
MLPN: Multi-layer perceptron network
VC: Vapnik-Chervonenkis
